# Relationship of systemic type I interferon activity with clinical phenotypes, disease activity, and damage accrual in systemic lupus erythematosus in treatment-naive patients: a retrospective longitudinal analysis

**DOI:** 10.1186/s13075-023-03010-0

**Published:** 2023-02-17

**Authors:** Kazusa Miyachi, Taro Iwamoto, Shotaro Kojima, Tomoaki Ida, Junya Suzuki, Takuya Yamamoto, Norihiro Mimura, Takahiro Sugiyama, Shigeru Tanaka, Shunsuke Furuta, Kei Ikeda, Kotaro Suzuki, Timothy B. Niewold, Hiroshi Nakajima

**Affiliations:** 1 Department of Allergy and Clinical Immunology, Graduate School of Medicine, Chiba University, 1-8-1 Inohana, Chuo-ku, Chiba, 260-8670 Japan; 2 Department of Medicine, Hospital for Special Surgery, New York, USA

**Keywords:** Systemic lupus erythematosus, Type I interferon, Clinical manifestation, Damage accrual, Biomarker

## Abstract

**Background:**

Systemic lupus erythematosus (SLE) is heterogeneous in organ involvement and disease severity, presenting a broad clinical phenotype. Systemic type I interferon (IFN) activity has been shown to be associated with lupus nephritis, autoantibodies, and disease activity in treated SLE patients; however, these relationships are unknown in treatment-naive patients. We aimed to determine the relationship of systemic IFN activity with clinical phenotypes, disease activity, and damage accrual in treatment-naive SLE patients before and after induction and maintenance therapy.

**Methods:**

Forty treatment-naive SLE patients were enrolled for this retrospective longitudinal observational study to examine the relationship between serum IFN activity and clinical manifestations of EULAR/ACR-2019 criteria domains, disease activity measures, and damage accrual. As controls, 59 other treatment-naive rheumatic disease patients and 33 healthy individuals were recruited. Serum IFN activity was measured by WISH bioassay and presented as an IFN activity score.

**Results:**

Treatment-naive SLE patients had significantly higher serum IFN activity than other rheumatic disease patients (score: 97.6 and 0.0, respectively, *p* < 0.001). High serum IFN activity was significantly associated with fever, hematologic disorders (leukopenia), and mucocutaneous manifestations (acute cutaneous lupus and oral ulcer) of EULAR/ACR-2019 criteria domains in treatment-naive SLE patients. Serum IFN activity at baseline significantly correlated with SLEDAI-2K scores and decreased along with a decrease in SLEDAI-2K scores after induction and maintenance therapy (*R*^2^ = 0.112, *p* = 0.034). SLE patients who developed organ damage (SDI ≥ 1) had higher serum IFN activity at baseline than those who did not (SDI = 0) (150.0 versus 57.3, *p*= 0.018), but the multivariate analysis did not detect its independent significance (*p* = 0.132).

**Conclusions:**

Serum IFN activity is characteristically high and is linked to fever, hematologic disorders, and mucocutaneous manifestations in treatment-naive SLE patients. Serum IFN activity at baseline correlates with disease activity and decreases in parallel with a decrease in disease activity after induction and maintenance therapy. Our results suggest that IFN plays an important role in the pathophysiology of SLE and that serum IFN activity at baseline may be a potential biomarker for the disease activity in treatment-naive SLE patients.

**Supplementary Information:**

The online version contains supplementary material available at 10.1186/s13075-023-03010-0.

## Introduction

Systemic lupus erythematosus (SLE) is a systemic autoimmune disease caused by dysregulation of the immune system and characterized by autoantibody production against double-stranded DNA (anti-dsDNA) and/or small nuclear RNA-binding proteins [1]. SLE is heterogeneous in organ involvement, disease severity, and immunopathogenesis [1] and is associated with the accumulation of irreversible organ damage, resulting in further damage and early mortality [2]. Then, the recent recommendations of treating-to-target strategies for the management of SLE would be expected to improve patient outcomes [3].

Accumulating evidence suggests that type I interferon (IFN) plays an important role in the pathogenesis of SLE [4]. High serum IFN activity is a heritable risk factor for SLE, as the familial accumulation of high serum IFN activity trait is observed in SLE families [5, 6], and some SLE susceptibility gene variants in the IFN pathway are gain-of-function in SLE patients [7–9]. High levels of serum IFN activity and increased expression of IFN-inducible genes (IIGs) in peripheral blood mononuclear cells (PBMCs) are associated with more severe diseases including lupus nephritis (LN) and the presence of SLE-associated autoantibodies [10–18]. In addition, anifrolumab, a monoclonal antibody to the IFN receptor, has recently been shown to be effective in moderate to severe SLE [19, 20]. However, these previous studies were mostly cross-sectional studies and reported the association between IFN activity and clinical and serological features of SLE in the treated patients. Thus, there have been no reports of the relationship between IFN activity and pathophysiological features of SLE in treatment-naive patients. Furthermore, the relationships between IFN activity and disease activity and damage accrual after induction and maintenance therapy have not yet been fully investigated. Therefore, to elucidate these issues, it is essential to conduct a longitudinal study in treatment-naive SLE patients to clarify the precise role of IFN in inducing clinical SLE. The main reason for the lack of longitudinal study data on serum IFN activity is due to the difficulty of serum IFN measurement by ordinary enzyme-linked immunosorbent assay (ELISA) [21, 22]. In addition, the determination of IIG expression in PBMCs is not sufficiently specific and quantitative, which also makes it difficult to compare the data between individuals at different time points [22].

In this retrospective longitudinal study, in order to determine the relationship of systemic IFN activity with clinical phenotypes, disease activity, and damage accrual in SLE, we measured serum IFN activity by WISH assay, a sensitive and quantitative bioassay for IFN [5, 22], and examined the link between serum IFN activity and 10 domains and their 21 individual items of the 2019 European League Against Rheumatism/American College of Rheumatology classification criteria for SLE (EULAR/ACR-2019 criteria) [23] in treatment-naive SLE patients. We also examined the relationship between serum IFN activity and the disease activity and organ damage accrual in the patients after induction and maintenance therapy.

## Patients and methods

### Study design and patients

This study is a retrospective longitudinal observational study enrolling 40 SLE patients who had had serum samples before their induction therapy. All the patients recruited fulfilled ≥ 4 of the American College of Rheumatology 1997 revised classification for SLE (ACR-1997 criteria) [24, 25]. The patient’s clinical data were evaluated just before the induction therapy (first assessment point) and after the induction and maintenance therapy (second assessment point), when patients were enrolled for the study at an arbitrary time during the maintenance therapy. The treatment-naive SLE patients had been managed in our university hospital from the disease onset and had been regularly followed up with its treatment every 2 to 3 months, and the median interval between the first and second assessment points was 7.1 years (IQR 2.8–9.6). IFN activities in the sera obtained at the first and second assessment points were measured simultaneously at the second assessment point.

In addition to 40 treatment-naive SLE patients, serum IFN activities were also analyzed in 59 treatment-naive patients with various rheumatic conditions (20 rheumatoid arthritis (RA), 21 systemic sclerosis (SSc), and 18 microscopic polyangiitis (MPA)) and 33 healthy individuals. Those treatment-naive patients with RA, SSc, and MPA were diagnosed according to previously described criteria [26–28]. All the non-lupus patients underwent induction immunotherapy, except for SSc patients, right after the blood collection. IFN measurement was done using these serum samples. All RA patients had ultrasound-proven multiple joint synovitis. Nineteen RA patients were treated with conventional synthetic disease-modifying anti-rheumatic drugs (csDMARDs) and one patient with biologic DMARDs right after the blood collection. Regarding MPA patients, all the patients underwent induction therapy with ≥ 0.5 mg/kg/day of prednisolone and rituximab.

### Laboratory and medical chart data collection

Baseline data such as the age of onset, sex, and observational period were collected. The baseline organ manifestations of SLE were assessed by both the ACR-1997 criteria and the EULAR/ACR-2019 criteria. The disease activity of SLE, at baseline and after induction and maintenance therapy, was scored according to the SLE Disease Activity Index 2000 (SLEDAI-2K) [29]. Antibody status of anti-nuclear antibody, anti-dsDNA, anti-Ro/La, anti-U1-RNP, anti-Sm antibodies, lupus anticoagulant, and anti-cardiolipin antibody (aCL) was also collected, and standard clinical cutoffs were used to define their positive results. The presence of lupus nephritis (LN) was defined as proteinuria ≥ 0.5 g/24 h or biopsy-proven nephritis compatible with SLE according to EULAR/ACR2019 criteria renal domain [23]. Subtypes of LN were confirmed by renal biopsy review according to the classification of the International Society of Nephrology/Renal Pathology Society (ISN/RPS) guidelines [30]. Drug information on induction and maintenance therapy was collected. Organ damage accrual was calculated by Systemic Lupus International Collaborating Clinics (SLICC) damage index (SDI) [31]. Disease flares were assessed according to the modified Safety of Estrogen in Lupus Erythematosus National Assessment (SELENA)-SLEDAI Flare Index without the physician global assessment (PGA) score [32].

### Serum IFN activity measurement

Serum IFN activity was measured by a bioassay using WISH epithelial cell line cells (WISH cells, ATCC #CCL-25) as described previously [5]. Briefly, healthy individuals and patients’ sera were incubated with WISH cells for 6 h to induce IIG transcription. Cells were then lysed for mRNA extraction, followed by cDNA synthesis. The expression levels of three canonical IIGs (i.e., IFN-induced protein with tetratricopeptide repeats 1 (IFIT1), myxovirus resistance 1 (MX1), and protein kinase R (PKR)) were measured by reverse transcriptase PCR. We then calculated an IFN activity score from the results of the three transcripts [5]. The amount of PCR product of the IIGs was normalized to the amount of product for the housekeeping gene GAPDH in the same sample. The relative expression of each of the three IIGs was calculated as a fold increase compared to its expression in WISH cells cultured with media alone. Then, the relative expressions of the three IIGs were summed and presented as an IFN activity score [5]. Pretreatment of the sera with anti-IFN-α and anti-IFN-β antibodies completely abrogates the IFN-induced gene expression observed in the assay. WISH cells are exquisitely sensitive to type I IFN and do not express type II IFN receptors or endosomal Toll-like receptors [5]. All the healthy individuals and SLE and other rheumatic disease patients’ IFN activity were measured by this bioassay.

### Statistical analysis

Statistical analysis was performed using the GraphPad Prism (version 9.3.1), SPSS software (version 28), and R statistical software (version 4.1.0). Principal component analysis (PCA) was performed using the prcomp command and factoextra (version 1.0.7) to identify the relationship between serum IFN activity and EULAR/ACR-2019 criteria domains. Continuous variables were expressed as medians and interquartile ranges (IQR) and compared by the Mann-Whitney *U* test. Categorical variables were described by the numbers and the percentages and compared by the chi-square test or Fisher’s exact test. A multi-group comparison was done by the Kruskal-Wallis test with Bonferroni correction. Correlation analysis was done by either Spearman’s rank-order correlation or linear regression. Logistic regression was performed to detect independent factors of organ damage accrual with an odds ratio and 95% confidence interval. *p* values < 0.05 were considered significant.

## Results

### Serum IFN activity is highly upregulated in treatment-naive SLE patients

**Fig. 1:**
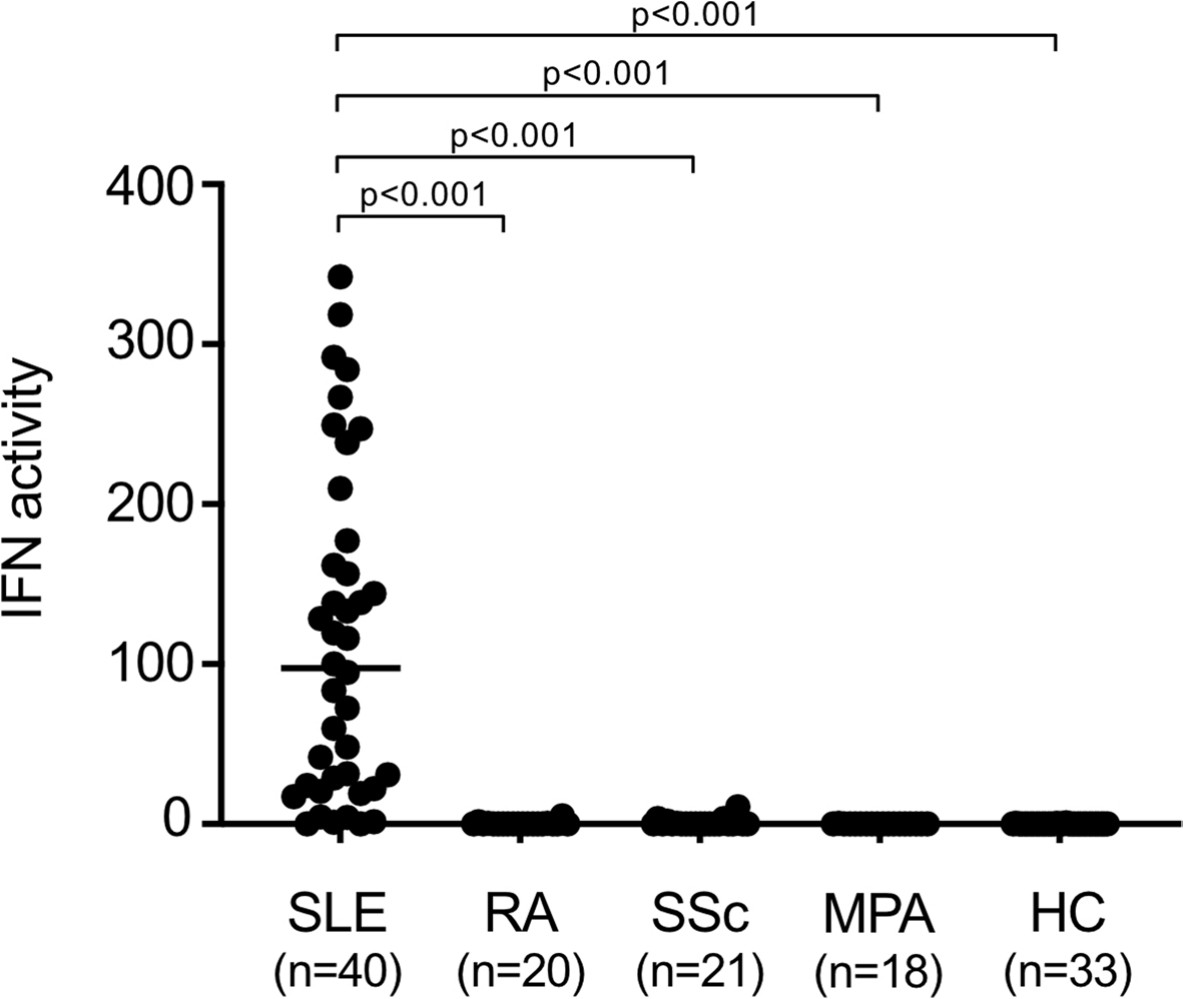
Serum IFN activities of treatment-naive SLE patients and other treatment-naive rheumatic disease patients. Serum IFN activities were measured in treatment-naive SLE patients (*n* = 40), other treatment-naive rheumatic disease patients (*n* = 59), and healthy individuals (*n* = 33) by WISH bioassay and were presented as an IFN activity score. None of these patients and healthy individuals has a history of glucocorticoid administration. The multi-group comparison was done by the Kruskal-Wallis test with Bonferroni correction. *p* values < 0.05 were considered significant. HC, healthy controls; IFN, type I interferon; MPA, microscopic polyangiitis; RA, rheumatoid arthritis; SLE, systemic lupus erythematosus; SSc, systemic sclerosis

We first examined the serum IFN activity in treatment-naive SLE patients and compared it with those of other treatment-naive rheumatic disease patients and healthy individuals (Fig. 1). Baseline characteristics of 40 treatment-naive SLE patients in this study are shown in Table 1. SLE patients showed significantly higher serum IFN activities (97.6 (22.8–173.3) (median (IQR), *n* = 40, *p* < 0.001), while patients with other rheumatic diseases and healthy individuals showed almost zero serum IFN activities (Fig. 1). Serum IFN activities of patients with RA (*n* = 20), SSc (*n* = 21), and MPA (*n* = 18) were 0.0 (0.0–0.1), 0.0 (0.0–0.3), and 0.0 (0.0–0.0), respectively. Serum IFN activity of healthy individuals (*n* = 33) was 0.0 (0.0–0.0), and the upper cutoff value (mean + 2SD) was calculated as 0.4. There were no significant differences in serum IFN activities between healthy individuals and patients with RA, SSc, or MPA (Fig. 1).

**Table 1:** Baseline characteristics of treatment-naive Japanese SLE patients

*n* = 40 patients	
Female, *n* (%)	37 (93)
Age of onset, median (IQR), years	31 (24–43)
Observation period, median (IQR), years	7.1 (2.8–9.6)
Numbers of ACR-1997 criteria domains, median (IQR), *n*	5 (4–6)
ACR-1997 criteria domains, *n* (%)	
Malar rash	19 (48)
Discoid rash	6 (15)
Photosensitivity	11 (28)
Oral ulcer	6 (15)
Arthritis	24 (60)
Serositis	8 (20)
Renal disorder	17 (43)
Neurologic disorder	3 (8)
Hematologic disorder	35 (88)
Immunologic disorder	40 (100)
ANA positive	40 (100)
Auto-antibody profile, *n* (%)	
Anti-dsDNA positive	36 (90)
Anti-U1-RNP positive	24 (60)
Anti-Sm positive	17 (43)
Anti-Ro/SS-A positive	27 (68)
Anti-La/SS-B positive	9 (23)
Lupus anticoagulant positive	12 (30)
Anti-cardiolipin positive	19 (48)
C3, median (IQR), mg/dl	42.5 (28.5–61.8)
C4, median (IQR), mg/dl	5.5 (3.3–11.8)
SLEDAI-2K, median (IQR), score	11 (8–16)
Induction dose of prednisolone, median (IQR), mg/day	40 (30–50)
Methylprednisolone pulse, *n* (%)	3 (8)
IVCY, *n* (%)	8 (20)
MMF, *n* (%)	7 (18)
HCQ, *n* (%)	5 (13)
CNIs, *n* (%)	4 (10)

*ACR-1997 criteria* American College of Rheumatology 1997 revised classification criteria for SLE, *ANA* antinuclear antibody, *C3/4* complement component 3/4, *CNIs* calcineurin inhibitors, *dsDNA* double-stranded DNA, *HCQ* hydroxychloroquine, *IVCY* intravenous cyclophosphamide, *MMF* mycophenolate mofetil, *RNP* ribonucleoprotein, *SLE* systemic lupus erythematosus, *SLEDAI-2K* SLE Disease Activity Index 2000, *Sm* Smith

### Serum IFN activity is associated with fever, hematologic disorders, and mucocutaneous manifestations in treatment-naive SLE patients

**Fig. 2:**
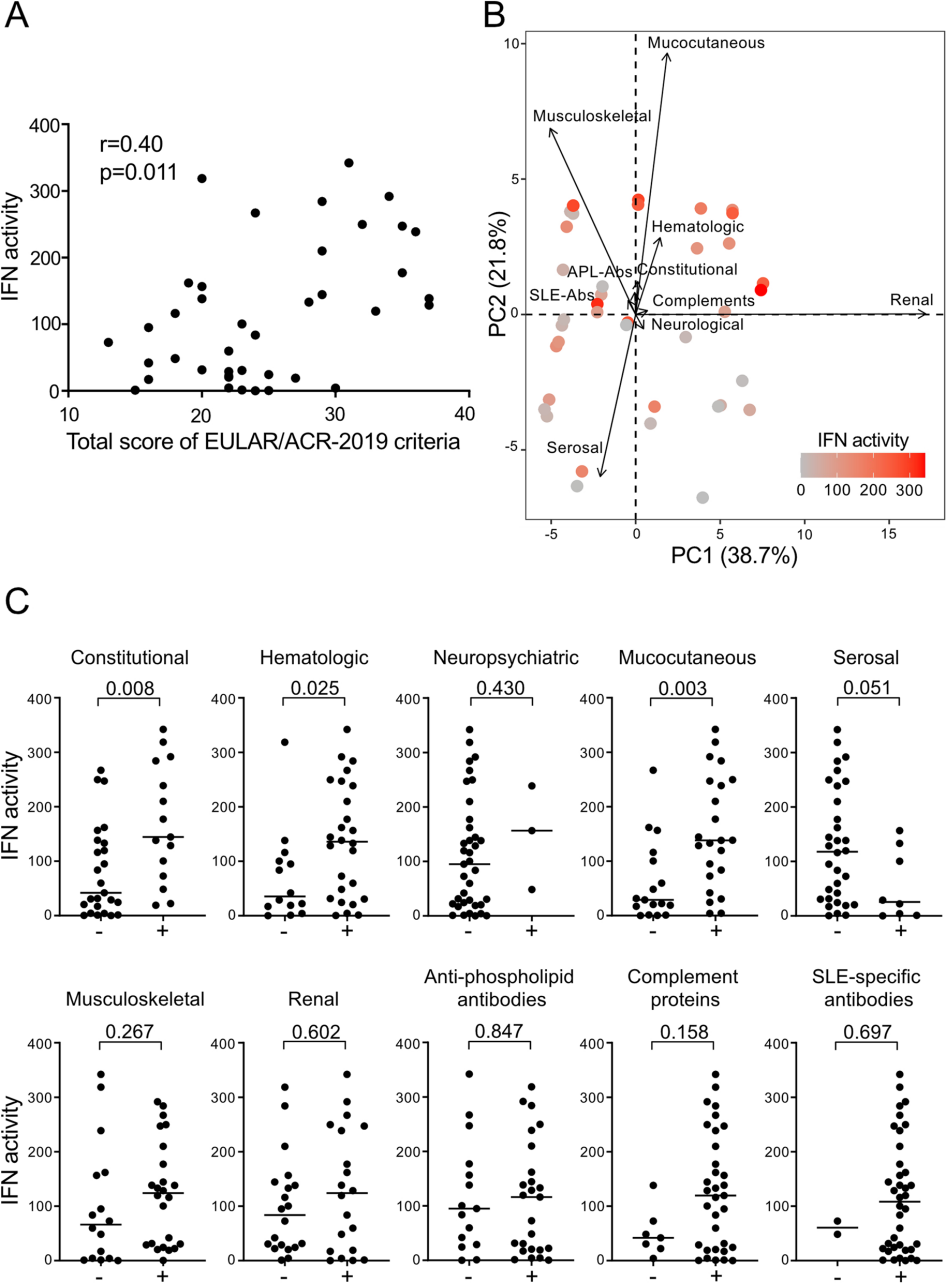
Associations between serum IFN activity and EULAR/ACR-2019 criteria domains in treatment-naive SLE patients. **A** Correlation between serum IFN activity and total score of the EULAR/ACR-2019 criteria was assessed in treatment-naive SLE patients (*n* = 40). **B** PCA was performed to examine the relationship between serum IFN activity and EULAR/ACR-2019 criteria domains. Each SLE patient is presented as a dot, with a gradation (gray to red) showing the level of serum IFN activity, and overlayed on the PCA. The arrow vector and length show the contribution ratio to the PCs. **C** Associations between serum IFN activity and each domain of the EULAR/ACR-2019 criteria were assessed by the Mann-Whitney test. *p* values < 0.05 were considered significant. Abs, antibodies; APL, anti-phospholipid; EULAR/ACR-2019 criteria, 2019 European League Against Rheumatism/American College of Rheumatology classification criteria for SLE; IFN, type I interferon

To determine the relationship of systemic IFN activity with clinical phenotypes in SLE, we examined the association between serum IFN activity and EULAR/ACR-2019 criteria domains in treatment-naive SLE patients. We found that serum IFN activity moderately and positively correlated with the total scores of the EULAR/ACR-2019 criteria (*r* = 0.40, *p* = 0.011, *n* = 40) (Fig. 2A). We first examined which EULAR/ACR-2019 criteria domains were tightly related to serum IFN activity in treatment-naive SLE patients by principal component analysis (PCA) (Fig. 2B). The cumulative contribution rate of PC1 and PC2 was 60.5% (Fig. 2B), suggesting that the symptoms of the enrolled patients were highly diverse. We calculated the correlation between serum IFN activity and each principal component and found that serum IFN activity did not correlate with PC1 (*r* = 0.14, *p* = 0.373) but did correlate with PC2 (*r* = 0.56, *p* < 0.001), which consisted of constitutional, hematologic, mucocutaneous, musculoskeletal, anti-phospholipid antibodies, complement proteins, and SLE-specific antibodies domains.

**Fig. 3:**
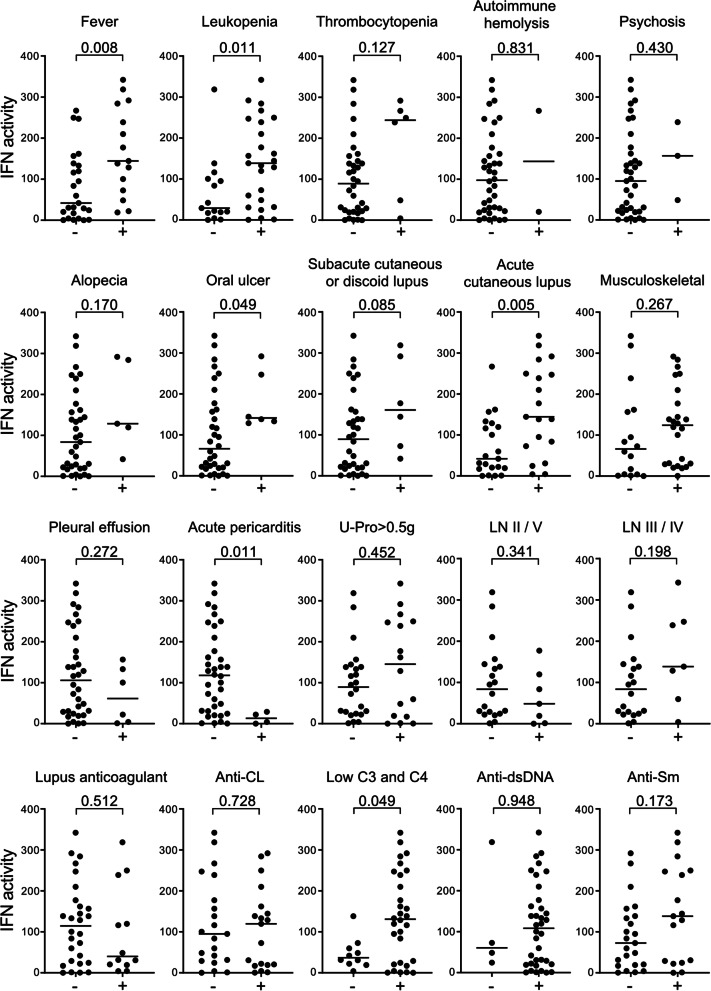
Detailed associations between serum IFN activity and EULAR/ACR-2019 criteria items of 10 domains in treatment-naive SLE patients. Associations between serum IFN activity and individual items that consist of EULAR/ACR-2019 criteria domains were assessed by the Mann-Whitney U test. *p* values < 0.05 were considered significant. C3/4, complement component 3/4; CL, cardiolipin; dsDNA, double-stranded DNA; EULAR/ACR-2019 criteria, 2019 European League Against Rheumatism/American College of Rheumatology classification criteria for SLE; IFN, type I interferon; LN, lupus nephritis; Sm, Smith; U-pro, urine protein

We then investigated the link between serum IFN activity and each domain of the EULAR/ACR-2019 criteria. Among 7 clinical domains and 3 immunological domains, SLE patients scored for the constitutional domain, hematologic domain, and mucocutaneous domain had significantly higher serum IFN activity than those who did not (*p* = 0.008, 0.025, and 0.003, respectively) (Fig. 2C). Among the 21 individual criteria items of 10 domains, patients who had fever, oral ulcer, acute cutaneous lupus, and leukopenia had significantly higher serum IFN activity than those who did not (*p* = 0.008, 0.049, 0.005, and 0.011, respectively) (Fig. 3). Serum IFN activity moderately and negatively correlated with white blood cell counts and platelet counts (*r* = − 0.43, *p* = 0.006 and *r* = − 0.54, *p* < 0.001, respectively).

In contrast, patients who were positive for the serosal domain tended to have lower serum IFN activity than patients negative for the domain (*p* = 0.051) (Fig. 2C) and patients who had acute pericarditis had significantly lower serum IFN activity than patients who did not (*p* = 0.011) (Fig. 3). There were no significant differences in serum IFN activities between the patients with and without renal domain and also among the subtypes of LN (Figs. 2C and 3).

Complements domain showed no significant differences in serum IFN activities between the patients with and without the domain (Fig. 2C), although the presence of one low complement item (i.e., low for both C3 and C4) was significantly associated with serum IFN activity (*p* = 0.049) (Fig. 3), but another low complement item (low for either C3 or C4) was not (*p* = 0.273, data not shown). Serum IFN activity moderately and negatively correlated with C3 and C4 concentrations (*r* = − 0.54, *p* < 0.001 and *r* = − 0.50, *p* = 0.001, respectively). Neither the SLE-specific antibody domain nor its items anti-dsDNA antibody and anti-Sm antibody positivity showed significant differences in serum IFN activities between the patients with and without the domain or items (Fig. 2C and Fig. 3).

### Decrease in serum IFN activity is associated with decreased disease activity after induction and maintenance therapy

**Fig. 4:**
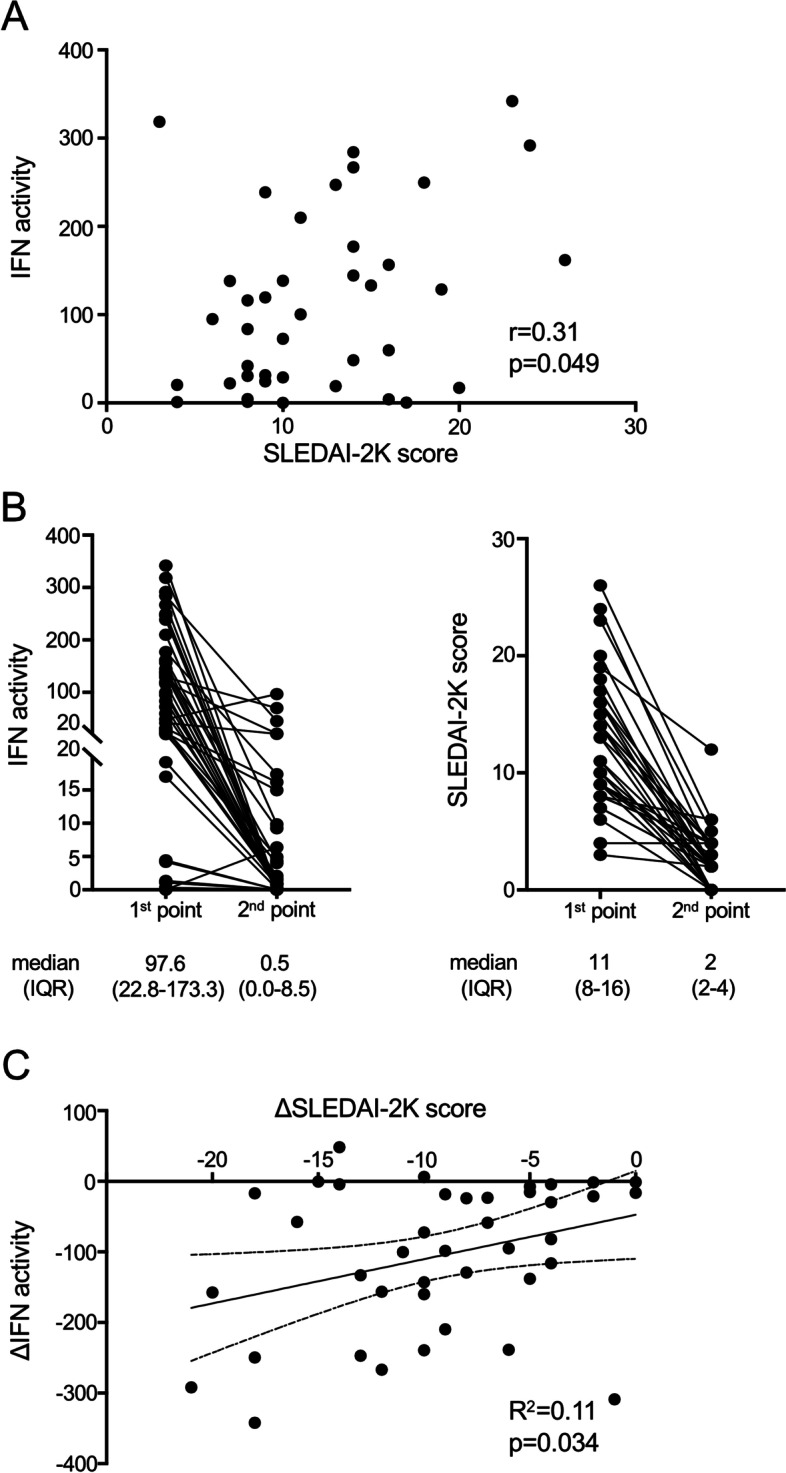
Relationship between serum IFN activity and SLEDAI-2K score before and after induction therapy. **A** Correlation between serum IFN activity and total score of SLEDAI-2K at baseline was assessed by Spearman’s rank-order correlation. **B** Changes in serum IFN activities and SLEDAI-2K scores before (1st point) and after (2nd point) the induction therapy are shown. **C** Correlation between changes in serum IFN activity and SLEDAI-2K score before and after induction therapy was examined by linear regression. IFN, type I interferon; SLEDAI-2K, SLE disease activity index 2000

We next examined the relationship between serum IFN activity and disease activity before and after induction therapy. The median observational period was 7.1 years between the first assessment point (before the induction therapy) and the second assessment point (after the induction and maintenance therapy). Serum IFN activity and SLEDAI-2K score at baseline (i.e., in the treatment-naive condition) were weakly and positively correlated (*r* = 0.31, *p* = 0.049) (Fig. 4A).

Serum IFN activity significantly decreased from 97.6 to 0.5 after induction and maintenance therapy (*p* < 0.0001) (Fig. 4B), while the SLEDAI-2K score also significantly decreased from 11 to 2 after induction and maintenance therapy (*p* < 0.0001), achieving low disease activity (SLEDAI-2K ≤ 4) (Fig. 4B). A decrease in IFN activity significantly correlated with the improvement of SLEDAI-2K score after induction and maintenance therapy (*R*^2^ = 0.112, *p* = 0.034) (Fig. 4C).

### Serum IFN activity, methylprednisolone pulse, and IVCY are associated with organ damage accrual in SLE patients

We then investigated the effect of systemic IFN activity on organ damage accrual in SLE patients. To do so, we examined the association between serum IFN activity at baseline and the characteristics of SLE patients with (SDI ≥ 1) and without (SDI = 0) organ damage. Univariate analysis revealed that patients who developed organ damage (SDI ≥ 1) had higher serum IFN activity at baseline than those who did not (150.0 versus 57.3, *p* = 0.018) (Table 2). Patients who had undergone methylprednisolone pulse therapy or intravenous cyclophosphamide (IVCY) pulse therapy were also significantly associated with damage accrual compared to those who did not develop organ damage (*p* = 0.020 and 0.018, respectively) (Table 2). No significant differences in sex, age, observation period, disease activity at baseline, the number of flares, induction dose of prednisolone, and anti-phospholipid syndrome-related antibody positivity were observed between these two groups.

**Table 2:** Comparison of characteristics between SLE patients with and without organ damage accrual stratified by SLICC damage index score

	SDI = 0 (*n* = 24)	SDI ≥ 1 (*n* = 16)	*p* value*
Female, *n* (%)	23 (96)	14 (88)	0.553
Age of onset, median (IQR), years	29 (24–41)	36 (24–46)	0.456
Observation period, median (IQR), years	7.9 (4.3–9.6)	5.7 (1.2–9.6)	0.308
SLEDAI-2K at baseline, median (IQR), score	10 (8–14)	14 (8–19)	0.207
Lupus nephritis^a^, *n* (%)	9 (38)	11 (69)	0.105
Lupus anticoagulant positive, *n* (%)	5 (21)	7 (44)	0.166
Anti-cardiolipin antibody positive, *n* (%)	11 (46)	8 (50)	0.999
Number of patients who had flare^b^, *n* (%)	14 (58)	8 (50)	0.748
Number of flares/patient, median (IQR), *n*	1 (0–1.75)	0.5 (0–1.75)	0.647
Serum IFN activity at baseline, median (IQR), score	57.3 (17.9–137.2)	150 (51.1–275.4)	0.018
Serum IFN activity at 2nd point, median (IQR), score	0.2 (0.0–6.0)	0.9 (0.0–15.5)	0.639
Induction dose of prednisolone, median (IQR), mg/day	38 (30–50)	43 (33–50)	0.273
Methylprednisolone pulse, *n* (%)	0 (0)	4 (25)	0.020
IVCY, *n* (%)	2 (8)	7 (44)	0.018
MMF, *n* (%)	8 (33)	8 (50)	0.339
HCQ, *n* (%)	5 (21)	7 (44)	0.166
CNIs, *n* (%)	11 (46)	9 (56)	0.748

*CNIs* calcineurin inhibitors, *HCQ* hydroxychloroquine, *IFN* type I interferon, *IVCY* intravenous cyclophosphamide, *MMF* mycophenolate mofetil, *SDI* Systemic Lupus International Collaborating Clinics (SLICC) damage index, *SLEDAI-2K* SLE Disease Activity Index 2000

^a^The presence of lupus nephritis (LN) was defined as proteinuria ≥ 0.5 g/24 h or biopsy-proven nephritis compatible with SLE according to the EULAR/ACR2019 criteria renal domain [23]

^b^Disease flares were assessed according to the modified Safety of Estrogen in Lupus Erythematosus National Assessment (SELENA)-SLEDAI Flare Index without the physician global assessment (PGA) score [29]

*p values were calculated with the chi-square test, Fisher’s exact test, or Mann-Whitney test. *p* values < 0.05 were considered significant

However, logistic regression detected the use of IVCY as an independent risk factor for developing organ damage (OR = 8.6, 95% CI 1.2–61.0, *p* = 0.031), but not serum IFN activity at baseline (*p* = 0.132) or a history of methylprednisolone pulse therapy (*p* = 0.999). Serum IFN activities at baseline were not significantly different between the patients with and without a history of IVCY therapy or methylprednisolone pulse therapy (data not shown).

## Discussion

We first showed that serum IFN activity was characteristically high in treatment-naive SLE patients compared with patients with other treatment-naive rheumatic diseases (RA, SSc, and MPA) or healthy individuals. Most previous studies regarding the role of IFN in the pathogenesis of SLE were cross-sectional in treated patients and measured IIG expression levels in PBMCs [12–15] due to the difficulty of serum IFN measurement by ordinary ELISA [21, 22]. In this study, we measured serum IFN activity by WISH bioassay, a sensitive and quantitative functional assay for IFN activity measurement using WISH cells, which are exquisitely sensitive to type I IFN [5]. To validate the accuracy of serum IFN measurement by WISH bioassay, we have additionally measured serum IFNα2a protein by S-PLEX immunoassay (Meso Scale Discovery, USA), a recently developed highly sensitive electrochemiluminescence immunoassay which could measure cytokines to fg/ml levels [33], from some of SLE patients in this study. We have found that the IFN score (by WISH bioassay) and the concentration of IFNα2a (by S-PLEX immunoassay) show a significant correlation (*r* = 0.96, *p* < 0.0001) (Additional file 1: Fig. S1). On the other hand, IIG expression in whole blood cells or PBMCs has also been used to evaluate IFN activity in SLE patients in many studies. An important caveat in determining IIG expression is that genes that are induced by type I IFN could sometimes be induced by other factors, for example, type II interferon. Furthermore, different individuals have varying proportions of immune cell types, and different immune cell types from the same blood sample express different expression levels of IIGs [22], and the proportions of immune cells may differ even in the same individual at different time points. In addition, the combination of IIGs used for estimating IFN activity varies among studies. Therefore, standardizing the titration of IFN activity using IIG expression levels in the whole blood or PBMCs is problematic; thus, it is difficult to compare the results of IIG expression levels between studies.

In addition, we found no significant increase in serum IFN activity in treatment-naive patients with RA, SSc, or MPA compared to that of healthy individuals (Fig. 1), which was in agreement with the findings by Hua et al. that there were no significant differences in plasma IFN activities between healthy individuals and RA patients in the assay using WISH cells and their expression of 5 IFNα-induced genes including our IFIT1, MX1, and PKR [34]. In contrast, a previous study showed that IIG expressions were upregulated in the whole blood of RA and SSc patients using Affymetrix microassays [35]. The difference in IFN values of RA and SSc patients compared to that of healthy individuals between the study [35] and ours might be due to their use of whole blood cells and different IIGs (IFI44, IFI44L, IFI27, RSAD2, and IFI6) that they had selected on the basis of increased IIGs among 5 diseases (SLE, RA, SSc, polymyositis, and dermatomyositis) [35], and those IIGs might be induced through the activation of the signaling pathways other than IFN pathways.

We next showed that serum IFN activity was significantly linked to fever, hematologic disorders (leukopenia), and mucocutaneous manifestations (acute cutaneous lupus and oral ulcer) of EULAR/ACR-2019 criteria domains in treatment-naive SLE patients (Figs. 2 and 3). IFN is well known to directly induce fever and also suppress the proliferation of pluripotent hematopoietic progenitor cells in the bone marrow, resulting in cytopenia [36, 37]. Our findings of the association of serum IFN activity with cutaneous manifestations are in agreement with the observation that the severity of cutaneous lesions positively correlated with IIG expression levels in PBMCs in SLE patients [38]. Furthermore, a single administration of a monoclonal antibody targeting BDCA2, a plasmacytoid dendritic cell (pDC)-specific receptor that inhibits the production of IFN, decreased the expression of IIGs in whole blood cells and reduced active cutaneous lesions in patients with SLE [39], while other studies showed that circulating pDC from SLE patients had impaired IFN production compared to that of healthy controls and the number of pDC was not associated with blood IIGs expression levels [40, 41]. Moreover, anifrolumab, a monoclonal antibody to the IFN receptor, has been shown to be very effective in cutaneous manifestations in SLE patients [19]. Therefore, interestingly, the associations of these manifestations with high serum IFN activity found in this study could be explained by the direct effect of IFN itself. On the other hand, previous studies did not detect a significant association of these manifestations with high systemic IFN activity in treated patients [14, 16]. In addition, because our results were based on exploratory analyses, these results must be tested in further confirmatory studies [42].

Although high serum IFN activity was observed in treatment-naive SLE patients, no significant association was found between serum IFN activity and the presence of LN or SLE-specific antibodies (Figs. 2 and 3). On the other hand, we have recently shown that serum IFN activity is significantly associated with class III/IV LN but not with class II/V LN in treated European-American SLE patients [18]. Some previous studies also found significant associations between high systemic IFN activity and LN in treated SLE patients [14, 15], but another study in the treated patients did not [16]. In addition to the differences in the prevalence and severity of LN among the ethnicities [43], the involvement of nephritis susceptibility genes which are not related to SLE susceptibility and more renal-specific that predispose specifically to LN might account for these different results in previous studies [44, 45].

We also demonstrated that serum IFN activity correlated with disease activity in treatment-naive SLE patients (Fig. 4A) and that serum IFN activity at baseline decreased along with a decrease in disease activity after induction and maintenance therapy (Fig. 4B). This is the first study to evaluate the changes in serum IFN activity at baseline and after induction and maintenance therapy. We found that the decrease in serum IFN activity significantly correlated with the improvement of SLEDAI-2K scores after induction and maintenance therapy (Fig. 4C). The decrease in serum IFN activity by treatment with glucocorticoids and immunosuppressants is probably due to the suppression of IFN production by pDCs, other immune cells, and non-hematopoietic cells. In addition, the decrease in autoantibodies by the treatment would also contribute to disrupting a positive feed-forward loop of autoantibody-immune complex-mediated IFN production in pDCs via endosomal nucleic acid-sensing Toll-like receptors in SLE [46]. Although the utility of measuring IFN activity (serum and IIGs in PBMC) as a biomarker for monitoring disease activity is still debated [47, 48], serum IFN activity at baseline may be useful for evaluating disease activity.

Damage accrual is also a great concern in the management of SLE [2, 49]. We found that SLE patients who developed organ damage had higher serum IFN activity at baseline and had a history of methylprednisolone pulse therapy or IVCY pulse therapy than those who did not (Table 2). However, the multivariate analysis only detected the history of IVCY pulse therapy as an independent risk factor of organ damage accrual in this population. Since many factors, including baseline characteristics (age, race, disease activity, and clinical phenotype), medication, and flares, could affect the damage accrual [31, 49], a medication effect (IVCY and glucocorticoid use) might have been significantly observed in this study. In addition, hydroxychloroquine (HCQ), a key drug for preventing damage accrual [49, 50], was approved in 2015 in Japan, and thus, the population of patients using HCQ was relatively low, which might have also affected the result of risk factors for damage accrual in this study. On the other hand, a previous study showed that high IIG expression in PBMCs was independently associated with LN, low complement levels, and SDI score in treated SLE patients by IIGs’ high/low categorical analysis [14]. Therefore, further investigation using a large prospective inception cohort is needed to elucidate the potential of baseline serum IFN activity as a biomarker for damage prediction in SLE patients.

The exact cellular source of high serum IFN activity in SLE patients is still unclear, although it has been thought that pDC and other immune cells contribute to systemic IFN activity [41]. It has recently been shown that local non-hematopoietic cells, such as keratinocytes in the skin and renal tubular cells, play an important role as IFN producers and are responsible for inducing local tissue inflammation [41]. Keratinocytes produced IFN (interferon-κ) to induce CD16^+^ dendritic cells into a proinflammatory phenotype in cutaneous lupus inflammation [40, 51]. On the other hand, few infiltrating pDCs with decreased IFN production were present in the lesions of cutaneous lupus inflammation [52]. Our previous study also showed that in kidney samples of inflamed lupus nephritis, infiltrating pDCs were limited and did not co-localize with IIG-expressing cells [18]. Therefore, immune cells such as conventional dendritic cells and CD16^+^ monocytes other than pDCs [52] could contribute to systemic IFN activity, and further investigations are needed to clarify the cellular source of high serum IFN activity in SLE.

The limitations of this study are its relatively small sample size, which may reduce the statistical powers of the results and its retrospective design. Although we were able to track patients in medical records for a certain period, including the disease onset, we could not sufficiently track the fluctuation of disease activities which could have affected the accrual of organ damage.

## Conclusions

We have shown that treatment-naive SLE patients have uniquely high serum IFN activity compared with patients with other rheumatic diseases or healthy individuals. High serum IFN activity is significantly linked to fever, hematologic disorders, and mucocutaneous manifestations of EULAR/ACR-2019 criteria domains in treatment-naive SLE patients. We have also shown that serum IFN activity at baseline correlates with disease activity and decreases in parallel with a decrease in disease activity after induction and maintenance therapy in SLE patients. Our results suggest that IFN plays an important role in the pathophysiology of SLE and that serum IFN activity at baseline may be a potential biomarker for the disease activity in treatment-naive SLE patients.

## Supplementary Information

**Additional file 1: Fig. S1.** Correlation of serum IFN measurement by WISH bioassay and S-PLEX immunoassay. Serum IFNα2a was additionally measured using the S-PLEX human IFNα2a kit according to the manufacturer’s instructions in 17 SLE patients who participated in this study. Correlation between serum IFN activity (WISH bioassay) and serum IFNα2a concentration (S-PLEX immunoassay) was assessed by Spearman’s rank-order correlation. *P* values<0.05 were considered significant.

## Data Availability

The datasets used and/or analyzed during the current study are available from the corresponding author upon reasonable request.

## Abbreviations

aCL: Anti-cardiolipin antibody
ACR-1997 criteria: American College of Rheumatology 1997 revised classification criteria for SLE
ANA: Antinuclear antibody
CNIs: Calcineurin inhibitors
C3/4: Complement component 3/4
dsDNA: Double-stranded DNA
EULAR/ACR-2019 criteria: 2019 European League Against Rheumatism/American College of Rheumatology classification criteria for SLE
HCQ: Hydroxychloroquine
IFN: Type I interferon
IIGs: IFN-inducible genes
IVCY: Intravenous cyclophosphamide
MMF: Mycophenolate mofetil
LN: Lupus nephritis
PBMCs: Peripheral blood mononuclear cells
pDC: Plasmacytoid dendritic cell
RNP: Ribonucleoprotein
SDI: Systemic Lupus International Collaborating Clinics (SLICC) Damage Index
SLE: Systemic lupus erythematosus
SLEDAI-2K: SLE Disease Activity Index 2000
