# Supplementary material for: Relationship of systemic type I interferon activity with clinical phenotypes, disease activity, and damage accrual in systemic lupus erythematosus in treatment-naive patients: a retrospective longitudinal analysis
Source: Arthritis Res Ther. 2023 Feb 17;25:26. doi: 10.1186/s13075-023-03010-0 (PMC9936752; doi:10.1186/s13075-023-03010-0)
Supplement: Supplementary file 1 — Additional file 1: Fig. S1. Correlation of serum IFN measurement by WISH bioassay and S-PLEX immunoassay. Serum IFNα2a was additionally measured using the S-PLEX human IFNα2a kit according to the manufacturer’s instructions in 17 SLE patients who participated in this study. Correlation between serum IFN activity (WISH bioassay) and serum IFNα2a concentration (S-PLEX immunoassay) was assessed by Spearman’s rank-order correlation. P values<0.05 were considered significant. [file 13075_2023_3010_MOESM1_ESM.docx]

**Supplemental Figure S1. Correlation of serum IFN measurement by WISH bioassay and S-PLEX immunoassay.**

Serum IFNα2a was additionally measured using the S-PLEX human IFNα2a kit according to the manufacturer’s instructions in 17 SLE patients who participated in this study. Correlation between serum IFN activity (WISH bioassay) and serum IFNα2a concentration (S-PLEX immunoassay) was assessed by Spearman’s rank-order correlation. P values<0.05 were considered significant.

**Supplemental Figure S1**

0.1

1

10

100

1000

1

10

100

1000

10000

100000

IFN activity

(WISH bioassay)

IFNα2a (fg/ml)

(S-PLEX immunoassay)

r=0.96

p<0.0001
